# Genetic burden across core genes of the PI3K–AKT–mTOR pathway is associated with susceptibility to microscopic polyangiitis: a Chinese cohort study

**DOI:** 10.3389/fimmu.2026.1807517

**Published:** 2026-04-23

**Authors:** Lizhen Li, Jing Yang, Chao Xue, Liepeng Chu, Huan Zhong, Jinlan Rao, Chang She, Lijuan Tan, Xi Fang, Shaoxia Shen, Yinyin Chen

**Affiliations:** 1 Department of Nephrology, Hunan Clinical Research Center for Chronic Kidney Disease, Hunan Provincial People’s Hospital, The First Affiliated Hospital of Hunan Normal University, Changsha, China; 2 Hunan Engineering Research Center for Prevention, Treatment and Rehabilitation of Kidney Diseases, Changsha, China; 3 Changsha Innovation Center for Integrated Diagnosis and Treatment Technology of Metabolic Dysfunction–Associated Kidney Diseases, Changsha, China; 4 Department of Nephrology, The Second Affiliated Hospital of Guangxi Medical University, Nanning, China

**Keywords:** autoimmune vasculitis, genetic burden, microscopic polyangiitis, MPO-ANCA, PI3K–AKT–mTOR pathway, sex differences

## Abstract

**Background:**

The PI3K–AKT–mTOR signaling pathway plays a central role in immune regulation and has been implicated in autoimmune diseases. However, the contribution of genetic variation within key components of this pathway to microscopic polyangiitis (MPA) remains incompletely understood.

**Methods:**

We conducted a genetic association study in a Chinese cohort including MPA patients and controls. Four single nucleotide polymorphisms (SNPs) within core genes of the PI3K–AKT–mTOR pathway (PIK3CA, AKT1, and MTOR) were analyzed. A cumulative genetic burden score was constructed by summing the number of risk alleles across loci. Participants were stratified into burden categories based on the distribution in controls. Logistic regression, trend analysis, and sensitivity analyses restricted to hospital-based controls were performed.

**Results:**

A high genetic burden within the PI3K-AKT-mTOR pathway was associated with increased susceptibility to MPA, with a significant linear trend across burden categories, whereas the intermediate burden group showed no significant association, suggesting a threshold-dependent effect. In sex−stratified analyses, associations appeared more evident among females, although a formal test for interaction did not indicate statistical significance. Analyses suggest a potential sex-related trend that warrants further investigation. Sensitivity analyses restricted to hospital-based controls yielded consistent results. Several common haplotypes spanning PIK3CA, AKT1, and MTOR were less frequent among patients, indicating potential protective effects. Pathway-level genetic burden was also associated with MPO-ANCA positivity. Single-variant analyses revealed generally concordant but modest effects.

**Conclusions:**

Genetic variation across selected core components of the PI3K--AKT--mTOR pathway may contribute to susceptibility to MPA. The observed patterns, including potential sex-related differences, should be interpreted cautiously and require validation in larger and independent cohorts. These findings highlight a potential pathway-level genetic architecture underlying MPA susceptibility.

## 1 Introduction

Microscopic polyangiitis (MPA) is a necrotizing small-vessel vasculitis strongly associated with antineutrophil cytoplasmic antibodies (ANCA), most commonly myeloperoxidase (MPO)-ANCA (1, 2). It represents a predominant subtype of ANCA-associated vasculitis (AAV) in East Asian populations (3). Despite advances in immunosuppressive therapy, disease relapse and progressive organ damage remain frequent (4), highlighting persistent gaps in our understanding of disease susceptibility and immunopathogenesis (2). Increasing evidence suggests that genetic factors contribute substantially to MPA risk (5), yet the molecular pathways shaping this susceptibility remain incompletely characterized.

The phosphoinositide 3-kinase (PI3K)–AKT–mechanistic target of rapamycin (mTOR) signaling pathway is a central regulator of immune cell activation (6), metabolism, and survival (6–8). This pathway integrates signals from cytokine receptors, antigen receptors (e.g., B cell and T cell receptors), and growth factors, thereby influencing neutrophil activation, antigen presentation, lymphocyte differentiation, and endothelial integrity (9–11)—processes that are directly relevant to ANCA-associated vasculitis (12). Experimental studies have implicated aberrant PI3K–AKT–mTOR signaling in inflammatory vascular injury and autoimmunity (13), and pharmacological modulation of mTOR has demonstrated immunoregulatory effects in immune-mediated diseases (14).

In our earlier work, we assessed the relationship between selected single-nucleotide polymorphisms in core genes of the PI3K–AKT–mTOR pathway, including *PIK3CA*, *AKT1*, and *MTOR*, and susceptibility to AAV. Although several variants showed suggestive associations, the analyses were restricted to isolated loci (15). Such single-variant strategies are inherently limited in their ability to capture the aggregate and coordinated effects of biologically connected genes within a shared signaling network (16). A pathway-based genetic approach may therefore provide a more informative framework for understanding disease susceptibility.

Sex represents an additional, clinically relevant modifier in MPA (17), as accumulating evidence indicates that males and females differ in clinical presentation, laboratory parameters, and histopathological features in ANCA-associated vasculitis (18, 19). Differences in incidence, immune responses, and clinical outcomes between males and females suggest that genetic effects may be context-dependent (20–22). Given that sex hormones and sex-specific immune regulation intersect with PI3K–AKT–mTOR signaling (23), it is plausible that genetic variation within this pathway exerts differential effects across sexes. Moreover, whether pathway-level genetic architecture contributes to ANCA serological heterogeneity (e.g., MPO-ANCA/MPO-ANCA positivity) remains unclear.

Accordingly, this study aimed to evaluate the association between PI3K–AKT–mTOR pathway genetic variation and MPA susceptibility using an integrative analytical strategy incorporating single-variant, pathway-level genetic burden, and haplotype analyses. Sex-stratified and MPO-ANCA–focused analyses were performed to assess effect modification and immunological relevance.

## 2 Materials and methods

### 2.1 Study population

**Figure 1:**
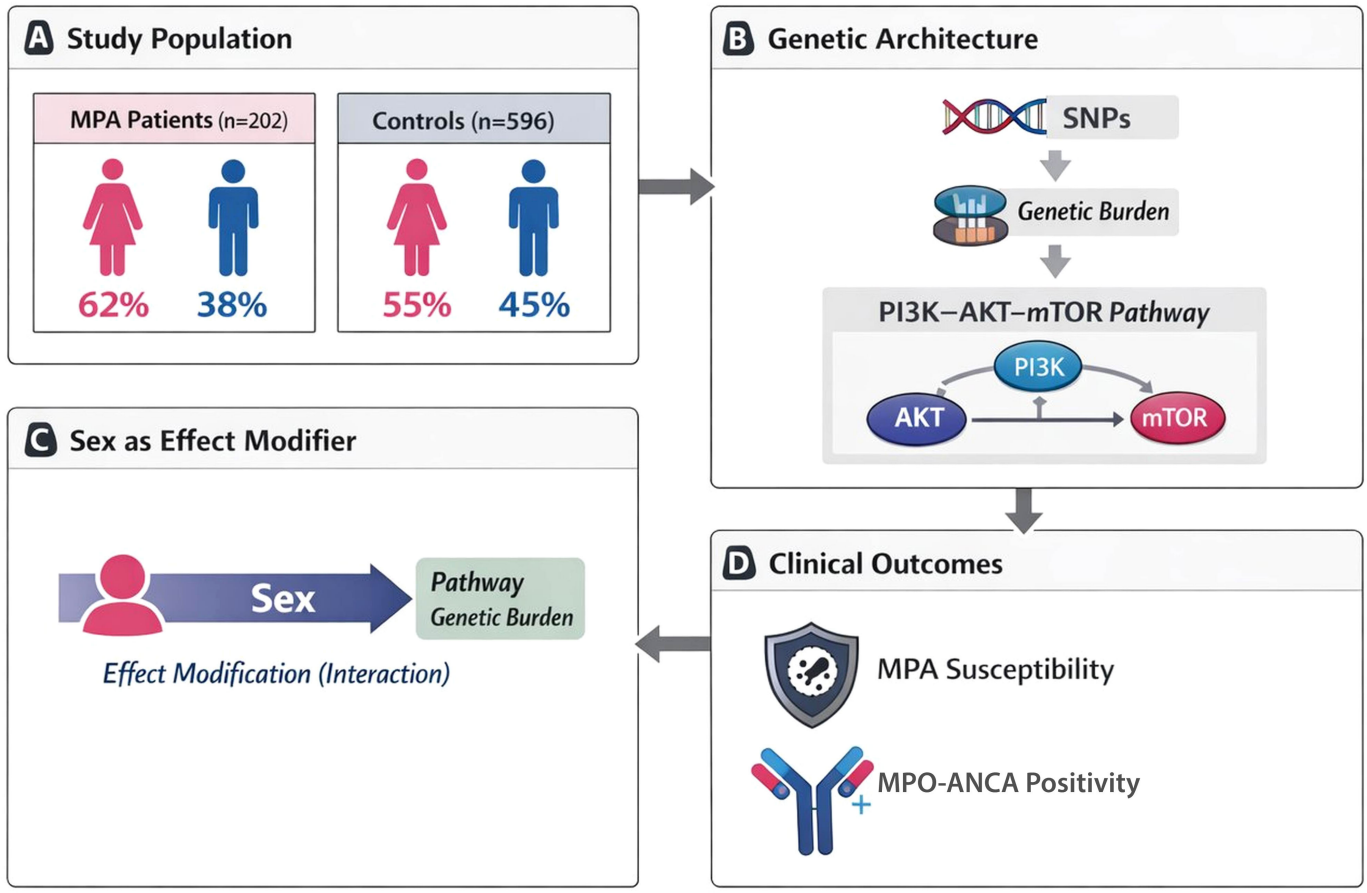
Overview of the study design and analytical framework. **(A)** Study population including 202 MPA patients and 596 controls. **(B)** Genetic architecture illustrating the progression from SNPs to cumulative genetic burden within the PI3K–AKT–mTOR pathway. **(C)** Sex as an effect modifier influencing pathway-level genetic burden. **(D)** Clinical outcomes including MPA susceptibility and MPO-ANCA positivity.

A total of 798 adults were enrolled in the present study, including 202 patients with microscopic polyangiitis (MPA) and 596 control participants (**Figure 1**). Patients with MPA were consecutively recruited from the Second Affiliated Hospital of Guangxi Medical University between September 2009 and October 2023. All patients fulfilled the diagnostic criteria for MPA according to the 2012 Chapel Hill Consensus Conference on the Nomenclature of Vasculitis. Individuals with a history of malignancy, active infection, or drug-induced vasculitis were excluded.

The control population comprised two independent sources. A total of 209 healthy controls were recruited from the same hospital during the same period, and an additional 387 unrelated Chinese individuals were obtained from the 1000 Genomes Project database (https://www.internationalgenome.org*)*. To enhance statistical power, the two control datasets were pooled after ensuring genetic homogeneity between them. To assess potential population stratification, genotype distributions between the two control sources were compared using chi-square tests (**Supplementary Table 1**). Three of four SNPs showed no significant differences after FDR correction, while the remaining SNP exhibited only a marginal difference unlikely to reflect meaningful stratification. Principal component analysis (PCA), performed using PLINK (v1.9) with standardized genotype data, demonstrated substantial overlap between hospital-based and 1000 Genomes controls, with no distinct clustering by sample source (**Supplementary Figure 1**). Given the sparse marker set, PCA was not intended to capture fine-scale population structure.

Among the MPA patients, 76 were male and 126 were female. MPO-ANCA positivity was detected in 139 patients (68.8%). The pooled control group consisted of 266 males and 330 females. Detailed demographic and clinical characteristics of the study population are shown in **Table 1**.

**Table 1:** Baseline characteristics of the study population.

Characteristic	Total (n = 798)	MPA (n = 202)	Controls (n = 596)	P value
Sex, n (%)				0.09
Female	456 (57%)	126 (62%)	330 (55%)	
Male	341 (43%)	76 (38%)	265 (45%)	
MPO-ANCA positive, n (%)	–	139 (68.8%)	–	–
Genotyped samples (%)				
PIK3CA	788 (98.7)	193	595	–
AKT1	798 (100)	202	596	–
MTOR	798 (100)	202	596	–

Continuous variables were compared using Student’s t-test; categorical variables were compared using the χ² test.

### 2.2 DNA extraction, sequencing, and genotype acquisition

Venous blood was collected from 411 participants enrolled at the study center. Genomic DNA was extracted using a commercial blood DNA extraction kit (Tiangen, Beijing, China) according to the manufacturer’s instructions. DNA concentration and purity were assessed using a NanoDrop 2000 spectrophotometer (Thermo Fisher Scientific, USA), with acceptable A260/A280 ratios ranging from 1.8 to 2.0.

Polymerase chain reaction (PCR) primers targeting *PIK3CA*/*AKT1*/*mTOR* were designed and synthesized by Sangon Biotech (Shanghai, China). PCR products were purified using AMPure XP magnetic beads, and fragment integrity was evaluated by agarose gel electrophoresis. Whole-genome sequencing was subsequently performed on the Illumina HiSeq X Ten platform (Illumina, USA).

Raw sequencing reads were processed using Cutadapt (version 1.2.1) to remove adapter sequences and PRINSEQ-lite (version 0.20.3) to filter low-quality reads. Clean reads were aligned to the human reference genome using the Burrows–Wheeler Aligner (BWA, version 0.7.13-r1126), and variant calling was conducted with SAMtools (version 0.1.18). In addition, genotype data from 387 Chinese individuals were retrieved directly from the 1000 Genomes Project and used as an external reference population.

### 2.3 Statistical analysis

All statistical analyses were performed using SPSS software (version 25.0) and the SNPStats web-based platform for genetic association analysis (https://www.snpstats.net/start.htm). Continuous variables were summarized as mean ± standard deviation, whereas categorical variables were expressed as counts and percentages. Differences in genotype distributions between groups were evaluated using chi-square tests.

Associations between genetic variants and MPA susceptibility were assessed using logistic regression models, with odds ratios (ORs) and 95% confidence intervals (CIs) calculated accordingly. Linkage disequilibrium patterns and haplotype structures were analyzed using SNPStats and Haploview software (version 4.1).

For each of the four SNPs, the risk allele was defined as the allele more frequently observed in MPA patients than in controls. A cumulative genetic burden score was calculated for each individual by summing the number of risk alleles across the four loci (each scored 0, 1, or 2), yielding a theoretical range of 0 to 8. Based on the score distribution among controls, three burden categories were defined using tertile cut-points: low burden (≤5 risk alleles), intermediate burden (6–7 risk alleles), and high burden (>7 risk alleles).

Sex-stratified analyses and SNP-by-sex interaction analyses were conducted to explore potential effect modification by sex.

To account for multiple testing, the Benjamini–Hochberg procedure was applied to control the false discovery rate (FDR). Correction was performed separately within each set of analyses addressing distinct biological questions: the primary burden analysis, the haplotype tests, the sex-stratified analyses, and the MPO-ANCA–related analyses. Both unadjusted and FDR-adjusted P values are reported. As a complementary approach, a global FDR correction combining all primary tests was also performed; the key findings remained essentially unchanged, with the exception of haplotype H4 (C–G–T–T), which showed a borderline association after global correction (raw P = 0.043, global FDR P = 0.058)(**Table 2**).

**Table 2:** Haplotype structure and associations within the PI3K–AKT–mTOR pathway.

Haplotype	Frequency	OR (95% CI)	P (raw)	P (FDR-BH)	global FDR P
H1: C–C–T–T	0.430	1.00	–	–	–
H2: T–C–T–T	0.170	0.73 (0.50–1.06)	0.096	0.110	0.110
H3: C–C–G–C	0.110	0.97 (0.59–1.61)	0.910	0.910	>0.99
H4: C–G–T–T	0.092	0.54 (0.30–0.98)	**0.043**	0.047*	0.058
H5: T–G–T–T	0.063	0.47 (0.23–0.93)	**0.031**	0.038*	0.048*
Global haplotype association p-value: 0.011*					

Only haplotypes with a frequency ≥ 3% are shown. FDR-BH correction was applied within haplotype-level tests only. Of note, under a more stringent global FDR correction combining all primary tests, haplotype H4 (C–G–T–T) showed a borderline association (raw P = 0.043, global FDR P = 0.058). *, P (FDR-BH) <0.05.

Bold values denote P_FDR < 0.05 (raw P values are also bolded when FDR < 0.05).

### 2.4 Ethical approval

This study was approved by the Ethics Committee of the Second Affiliated Hospital of Guangxi Medical University (approval numbers: 2018 KY-0100 and 2024 KY-0782) and conducted in accordance with the principles of the Declaration of Helsinki. Written informed consent was obtained from all participants prior to enrollment.

## 3 Results

### 3.1 Demographic and clinical characteristics of the study cohort

The present study included 202 patients diagnosed with MPA and 596 unrelated healthy controls, yielding a total sample size of 798 participants (**Table 1**). The proportion of female subjects was marginally higher in the control group than in the MPA group (62% vs. 55%), although this difference did not reach statistical significance (χ² test, P = 0.09). Among MPA patients, 139 individuals (68.8%) were positive for MPO-ANCA.

Genotyping completion rates were high across all loci, exceeding 98% for *PIK3CA* and reaching 100% for *AKT1* and *mTOR*. No notable imbalance in genotyping success was observed between cases and controls, supporting the reliability of downstream genetic analyses.

### 3.2 Increased PI3K–AKT–mTOR pathway genetic burden is associated with elevated MPA risk

PCA demonstrated substantial overlap between hospital-based and 1000 Genomes controls, with no evidence of clustering by sample source (**Supplementary Figure 1**).

To investigate the cumulative contribution of genetic variants within the PI3K–AKT–mTOR signaling cascade, a pathway-based genetic burden score was generated and categorized into tertiles (**Table 3**). Individuals classified in the high-burden category demonstrated a significantly increased susceptibility to MPA compared with those in the low-burden reference group (OR = 1.98, 95% CI 1.25–3.12, P = 0.003). This association remained robust after correction for multiple testing using the Benjamini–Hochberg method (P_FDR = 0.004).

**Table 3:** Association between PI3K–AKT–mTOR pathway-level genetic burden and risk of microscopic polyangiitis.

Genetic burden	MPA (n%)	Controls (n%)	OR (95% CI)	P (raw)	P (FDR-BH)
Low (reference)	52 (19.7%)	212 (80.3%)	1.00	–	–
Intermediate	92 (24.6%)	282 (75.4%)	1.33 (0.91–1.95)	0.145	0.145
High	49 (32.4%)	100 (66.6%)	1.98 (1.25–3.12)	**0.003**	0.004**
P for trend (overall)		0.004**		

Odds ratios were estimated using logistic regression adjusted for sex. False discovery rate was controlled using the Benjamini–Hochberg method at the pathway-level. **, P (FDR-BH) <0.01.

Bold values denote P_FDR < 0.05 (raw P values are also bolded when FDR < 0.05).

In contrast, participants in the intermediate-burden group showed only a modest, non-significant increase in disease risk (OR = 1.33, 95% CI 0.91–1.95, P = 0.145). These results suggest a threshold-dependent effect, whereby a higher accumulation of pathway-related risk alleles confers a markedly increased likelihood of developing MPA.

A linear trend test across the three burden categories (low, intermediate, high) was performed using ordinal coding (0, 1, 2). A significant dose−response relationship was observed in the overall analysis (P = 0.004) and in females (P = 0.013), supporting a graded association between increasing genetic burden and MPA risk. In males, the trend did not reach significance (P = 0.129), consistent with the limited statistical power in this subgroup. When modeled as a continuous variable, the burden score remained significantly associated with MPA risk (OR = 1.18, 95% CI 1.05–1.33, P = 0.006).Sensitivity analyses restricted to the hospital-based control group (n = 209) yielded consistent results. The high-burden group remained significantly associated with increased MPA risk (OR = 2.19, 95% CI 1.26–3.81, P = 0.005), with a significant linear trend across burden categories (P = 0.006) (**Supplementary Table 7**).

### 3.3 Haplotype-based analysis identifies protective genetic configurations with pronounced female specificity

Haplotype reconstruction incorporating four polymorphisms across the PI3K–AKT–mTOR pathway revealed several common haplotypes with differential associations with MPA (**Table 2**). Two haplotypes (C–G–T–T (H4) and T–G–T–T (H5)) were less frequent among MPA patients and showed associations with reduced disease risk. Under the primary stratified FDR correction, both haplotypes reached statistical significance (H4: OR = 0.54, P_FDR = 0.047; H5: OR = 0.47, P_FDR = 0.038). However, under a more stringent global FDR correction, the association for H4 became borderline (global FDR P = 0.058) and was thus interpreted as suggestive, whereas H5 remained significant (global FDR P = 0.048).

**Figure 2:**
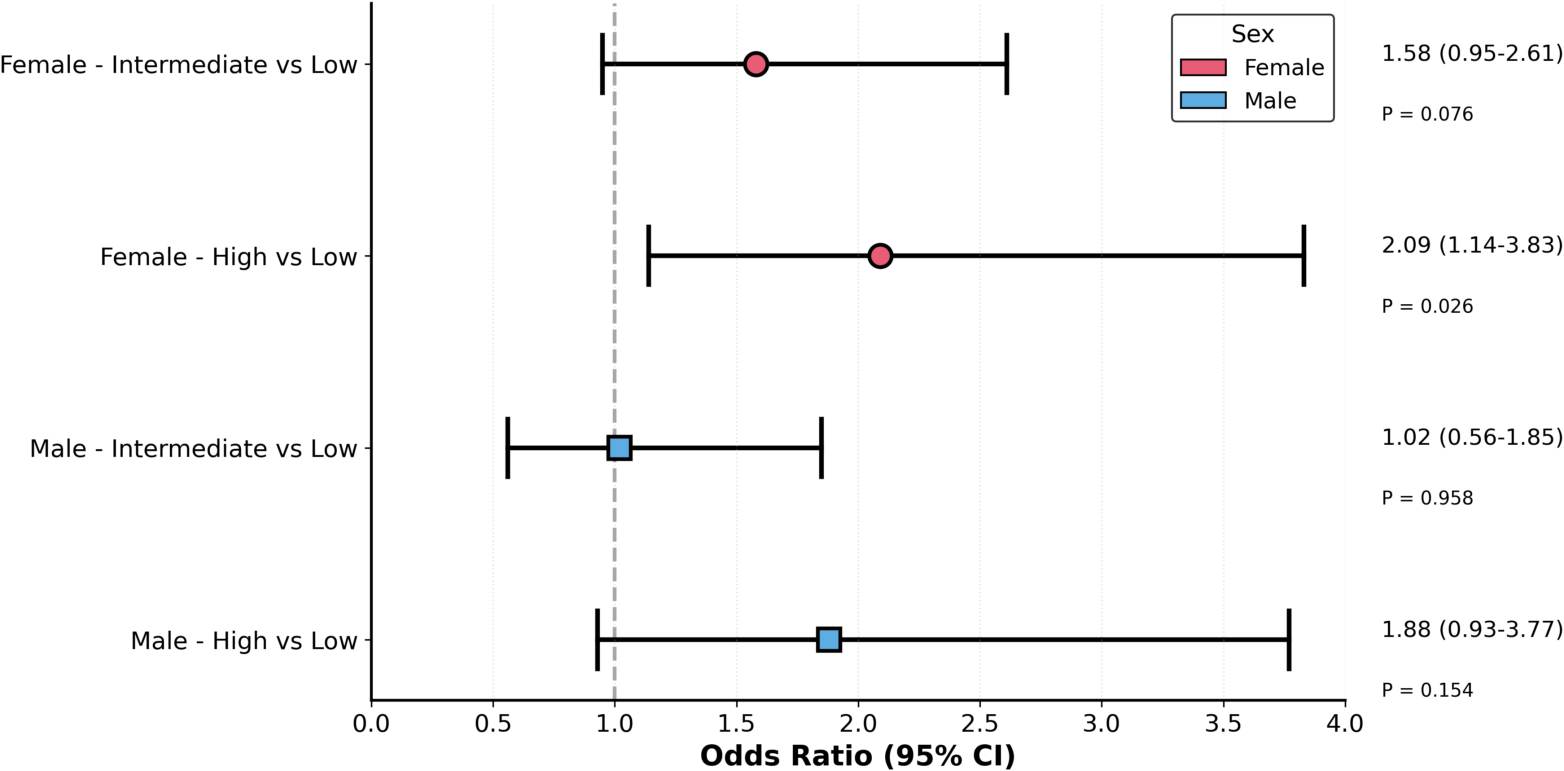
Female-driven haplotype architecture within the PI3K–AKT–mTOR pathway. Haplotype analysis of PI3K–AKT–mTOR pathway polymorphisms in relation to MPA risk. Common haplotypes (frequency ≥ 3%) were constructed and analyzed in the overall population and after stratification by sex. A significant global haplotype association was observed in females but not in males. Effect estimates were obtained from logistic regression models with false discovery rate adjustment.

Sex-stratified haplotype analysis revealed a striking divergence between females and males (**Figure 2**). A highly significant global association was detected in female participants (global P = 4.5×10^-4^), whereas no evidence of a comparable association was observed in males. These findings indicate that the haplotypic structure of the PI3K–AKT–mTOR pathway contributes predominantly to disease susceptibility in women.

### 3.4 Sex-dependent effects of pathway-level genetic burden on MPA susceptibility

**Figure 3:**
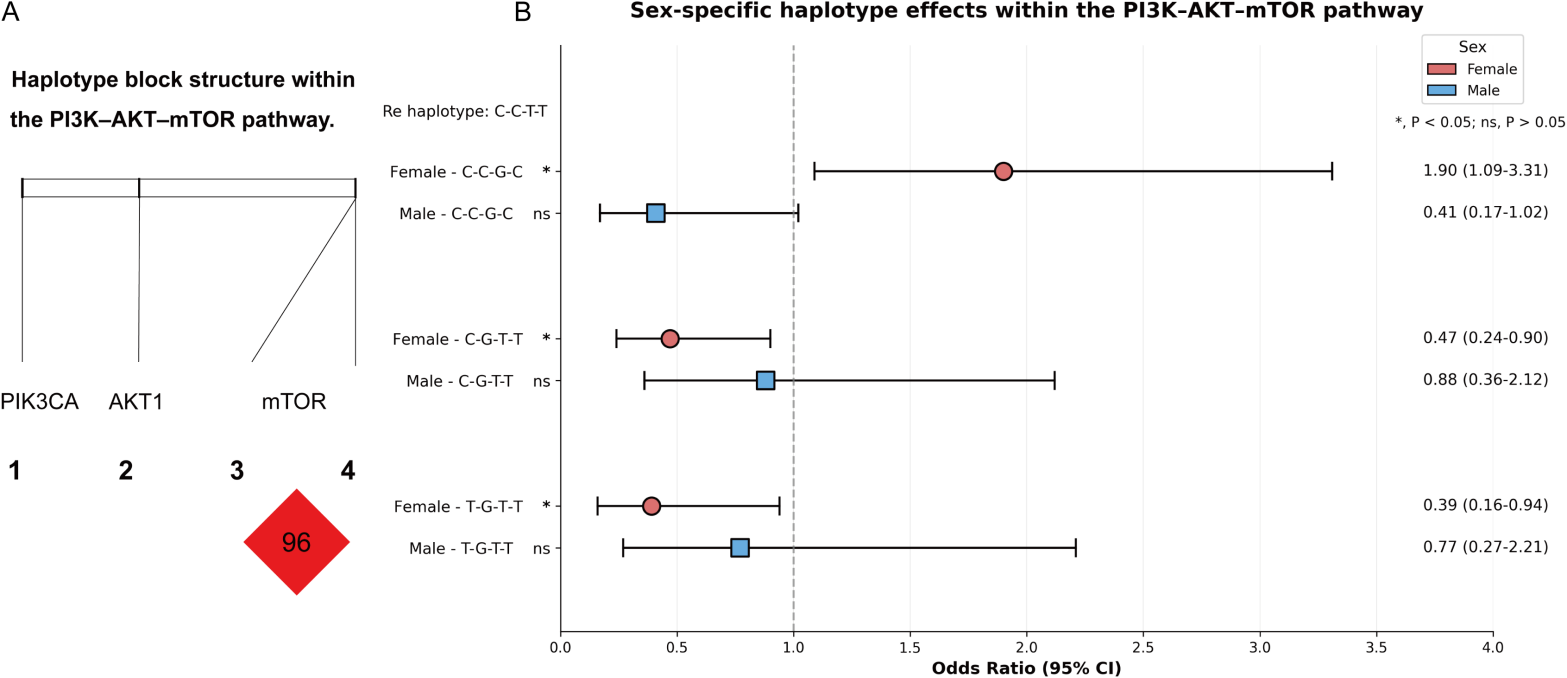
Sex-specific haplotype effects within the PI3K–AKT–mTOR pathway. **(A)** Haplotype block structure across PIK3CA, AKT1, and MTOR genes. **(B)** Forest plot showing sex-stratified associations between haplotypes and MPA risk, with odds ratios and 95% confidence intervals for females and males.

In light of the sex-stratified haplotype findings, pathway-level genetic burden was analyzed separately in female and male participants (**Table 4**; **Figure 3**). Among females, individuals classified in the intermediate burden group did not exhibit a statistically significant difference in MPA risk compared with the low-burden reference category (OR = 1.58, 95% CI 0.95–2.61). By contrast, females carrying a high pathway-level genetic burden showed a significantly increased risk of MPA (OR = 2.09, 95% CI 1.14–3.83), which remained significant after adjustment for multiple testing (P_FDR = 0.026).

**Table 4:** Sex-stratified burden analysis.

Sex	Pathway Burden	OR (95% CI)	P (raw)	P (FDR-BH)	Interaction P
Female	Low	1.00	–	–	0.716
	Intermediate	1.58 (0.95-2.61)	0.076	0.076	
	High	2.09 (1.14-3.83)	**0.017**	0.026*	
Male	Low	1.00	–	–	
	Intermediate	1.02 (0.56-1.85)	0.958	0.958	
	High	1.88 (0.93-3.77)	0.077	0.154	

Interaction P refers to the sex × High burden interaction term. Male non-significance is likely due to insufficient power (*post-hoc* power = 55% at observed OR = 1.88). *, P<0.05.

Bold values denote P_FDR < 0.05 (raw P values are also bolded when FDR < 0.05).

In males, neither the intermediate nor the high burden groups were significantly associated with MPA susceptibility. Although a trend toward increased risk was observed among males in the high-burden group (OR = 1.88, 95% CI 0.93–3.77), this association did not reach statistical significance in either the unadjusted or FDR-corrected analyses. To evaluate whether the effect of genetic burden differed by sex, a sex-by-burden interaction term was included in the logistic regression model. The interaction was not statistically significant (P = 0.716), indicating limited evidence for effect modification in the current sample. Accordingly, the observed sex-specific patterns should be interpreted as suggestive rather than definitive. *Post-hoc* power analysis further indicated limited statistical power in the male subgroup (~55% at the observed effect size), suggesting that the lack of statistical significance may reflect insufficient power rather than a true absence of association (**Supplementary Figure 2**).

### 3.5 Association between pathway genetic burden and MPO-ANCA positivity in MPA patients

**Figure 4:**
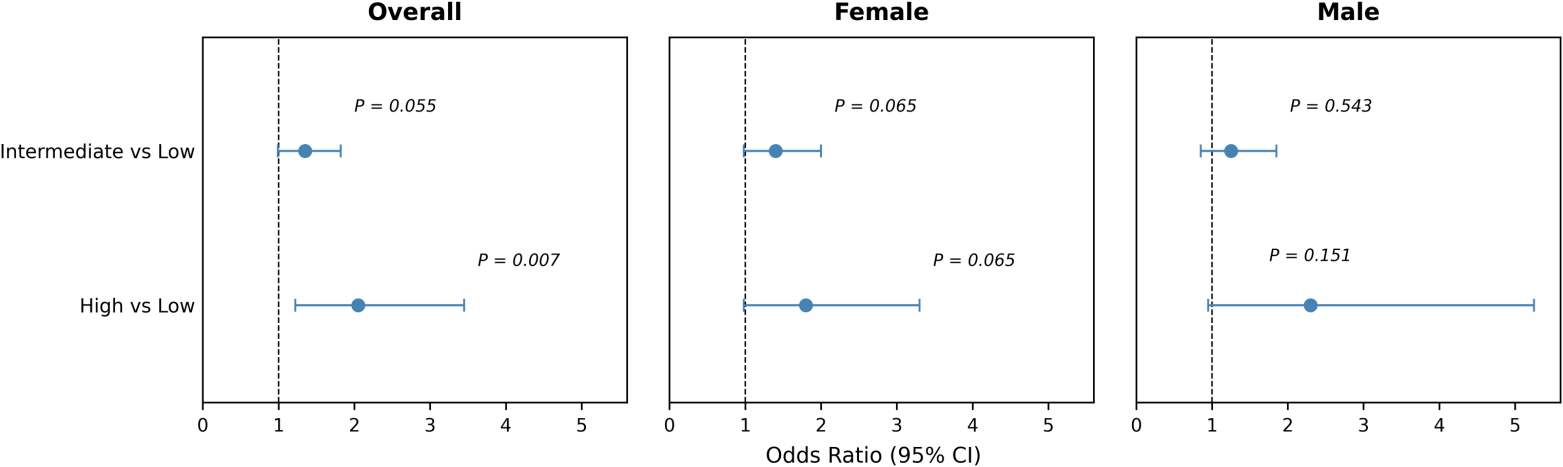
Sex-dependent association between pathway genetic burden and MPO-ANCA positivity. Relationship between pathway-level genetic burden and MPO-ANCA positivity in MPA patients. Associations were assessed using logistic regression with false discovery rate adjustment. Sex-specific patterns are shown.

To assess whether pathway-level genetic architecture influences immunological phenotypes within MPA, analyses were conducted among patients stratified by MPO-ANCA status (**Table 5**; **Figure 4**). Compared with patients in the low-burden group, those with a high genetic burden showed a significantly higher likelihood of MPO-ANCA positivity (OR = 2.07, 95%CI, 1.22–3.54, P = 0.007), which remained significant following FDR correction.

**Table 5:** Association between pathway-level genetic burden and MPO-ANCA positivity.

Pathway genetic burden	OR (95% CI)	P
Low (reference)	1.00	–
Intermediate	1.56 (0.99-2.45)	0.055
High	2.07 (1.22-3.54)	0.007**

Analyses were restricted to MPA patients. FDR-BH correction was applied within MPO-ANCA–related pathway-level tests. **, P (FDR-BH) <0.01.

Bold values denote P<0.05.

Patients in the intermediate-burden group demonstrated a borderline association with MPO-ANCA positivity (OR = 1.56, P = 0.055), suggesting a potential burden-dependent trend. These findings indicate that cumulative variation within the PI3K–AKT–mTOR pathway is linked not only to disease susceptibility but also to serological characteristics of MPA.

### 3.6 Concordant evidence from single-variant and sex-stratified analyses

Single-variant analyses of PI3K–AKT–mTOR pathway polymorphisms were consistent with the pathway-level findings. Significant associations with MPA risk were observed for variants in PIK3CA (rs1607237), AKT1 (rs2498786), and MTOR (rs1057079) under multiple genetic models after FDR correction. Sex-stratified and SNP-by-sex interaction analyses indicated that these associations were more pronounced in females than in males, as detailed in the supplementary tables (**Supplementary Table 2**-**6**).

## 4 Discussion

The present analyses suggest that coordinated genetic variation within the PI3K–AKT–mTOR signaling pathway contributes to susceptibility to microscopic polyangiitis, with effects that vary by sex and are associated with MPO-ANCA positivity. Taken as a whole, our findings suggest that susceptibility to MPA is better captured by the combined effects of genetic variation across a functionally connected immune signaling pathway, rather than by single loci evaluated in isolation (24).

At the pathway level, an increased genetic burden was associated with MPA primarily at the highest burden category, whereas intermediate levels of variation were not significantly linked to disease risk. This threshold-dependent association suggests that partial perturbation of PI3K–AKT–mTOR signaling may be insufficient to disrupt immune homeostasis. Such a pattern aligns with experimental evidence indicating that PI3K–AKT–mTOR signaling functions as a key regulator of activation thresholds in innate immune cells (6). Consistent with this concept, experimental studies have shown that PI3K–Akt signaling is required for ANCA-primed neutrophil activation and oxidative burst, which represent early pathogenic events in necrotizing small-vessel vasculitis (25–27). In this context, relatively modest functional effects at individual loci are unlikely to be sufficient on their own but may become biologically relevant when aggregated across the pathway, promoting persistent inflammatory activation and vascular damage.

Haplotype analyses further highlighted the importance of coordinated genetic architecture within the PI3K–AKT–mTOR pathway. Several common haplotypes spanning *PIK3CA*, *AKT1 (*15), and *MTOR (*28) were less frequent among patients with MPA, suggesting that specific allelic configurations may help preserve signaling balance within this pathway. Notably, such coordinated effects are unlikely to be detected through single-variant analyses alone, underscoring the additional information gained from haplotype-based approaches in pathway-oriented genetic studies.

One of the most striking observations in this study was the pronounced sex specificity of the genetic associations, which were predominantly observed in female participants (29). Sex-dependent differences in immune regulation are well recognized, particularly in pathways governing T cell help, B cell activation, and autoantibody production (3). These immunological processes intersect with PI3K–AKT–mTOR signaling at multiple levels, providing a biologically plausible context in which inherited variation within this pathway may exert stronger effects in females. In this biological setting, even subtle inherited perturbations in PI3K–AKT–mTOR signaling may have a greater likelihood of tipping immune responses toward enhanced activation and loss of tolerance.

Several lines of evidence may help explain the female-predominant pattern observed in our study. One potential explanation for the pronounced female-specific genetic associations observed in our study lies in well-characterized sex differences in immune signaling and regulation. Estrogen receptors, expressed broadly on immune cells including lymphocytes, can engage non-genomic signal transduction cascades in addition to classical transcriptional programs. In particular, estrogens have been shown to activate the phosphoinositide 3-kinase (PI3K)–AKT axis through membrane-associated estrogen receptors or ligand-independent interactions with signaling complexes, thereby enhancing downstream survival and activation signals in lymphoid cells, including B cells and T cells. This mode of crosstalk with PI3K signaling has been implicated in promoting cellular survival, proliferation, and functional activation in adaptive immune populations (30). Concurrently, estrogen enhances mechanistic target of rapamycin complex 1 (mTORC1) activity in B cells, a pathway integral to class-switch recombination and plasma cell differentiation, processes that underpin robust antibody and autoantibody responses (21).

Genetic and epigenetic differences linked to sex chromosomes provide an additional layer of immune regulation that may intersect with PI3K–AKT–mTOR signaling. Several immune-related genes encoded on the X chromosome, such as TLR7, TLR8, and FOXP3, are known to escape X-chromosome inactivation, leading to higher expression in females relative to males (31). These genes influence innate and adaptive immune activation thresholds—TLR7 and TLR8 enhance endosomal pattern recognition and type I interferon responses, while FOXP3 modulates regulatory T cell differentiation and function—thereby collectively lowering the threshold for immune activation in females (31). The integration of hormone-mediated signaling with dosage effects of X-linked immune genes thus offers a biologically plausible framework in which inherited variation in PI3K–AKT–mTOR pathway components could exert sex-biased effects on vasculitis susceptibility and MPO-ANCA production.

Additional support for the involvement of this pathway in vasculitic autoimmunity comes from monogenic disorders of immune regulation. Gain-of-function variants in PIK3CD or PIK3R1, as observed in activated PI3Kδ syndrome, have been associated with ANCA vasculitis and systemic immune dysregulation (32–34). Importantly, clinical improvement following PI3Kδ or mTOR inhibition in this setting highlights a direct link between dysregulated PI3K–AKT–mTOR signaling, breakdown of immune tolerance, and vascular inflammation (35–37), thereby providing translational context for the genetic associations observed in the present study.

In addition to disease susceptibility, pathway-level genetic burden was associated with MPO-ANCA positivity among patients with MPA. MPO-ANCA production reflects coordinated interactions among neutrophils, antigen-presenting cells, and autoreactive B cells, all of which are regulated by PI3K–AKT–mTOR signaling (38–40). Prior functional and transcriptomic studies in ANCA-associated disease have implicated this pathway in neutrophil activation programs relevant to autoantigen exposure and downstream immune amplification (41). Together, these results indicate that inherited variation within the PI3K–AKT–mTOR pathway may shape not only susceptibility to vasculitis but also key serological features such as MPO-ANCA positivity.

Single-variant analyses of *PIK3CA*, *AKT1*, and *MTOR* were generally concordant with the pathway-level findings, particularly in females, although individual effect sizes were modest. While the contribution of any single variant was limited, these modest effects accumulated meaningfully in the pathway−level burden analysis, suggesting that aggregate variation across the PI3K–AKT–mTOR pathway—rather than isolated loci—may be more relevant to disease−relevant signaling dysregulation. This observation reinforces the concept that cumulative and interactive genetic effects may provide a more informative framework for understanding complex immune-mediated diseases than isolated polymorphisms alone (42, 43).

Overall, our data support a model in which aggregate genetic variation across the PI3K–AKT–mTOR pathway contributes to MPA susceptibility and MPO-ANCA serological status in a sex-dependent manner (44). A pathway-oriented and sex-aware analytical perspective may therefore be particularly valuable for dissecting genetic contributions to clinical and immunological heterogeneity in ANCA-associated vasculitis.

### Study limitations and perspectives

Several limitations merit consideration. First, the primary analysis combined hospital-based controls with 1000 Genomes data lacking age information; thus, models were adjusted for sex only. Sensitivity analyses restricted to the hospital cohort, with adjustment for both age and sex, yielded consistent results. Second, all participants were of Chinese ancestry (mainly southern China). As allele frequencies and linkage disequilibrium patterns differ across populations, replication in European, Japanese, and other East Asian cohorts is needed.

Third, only four polymorphisms in three genes (PIK3CA, AKT1, MTOR) were analyzed. Other pathway components (e.g., PTEN, PDK1, TSC1/TSC2, RPTOR) were not included. Importantly, the limited number of SNPs restricts comprehensive coverage of the pathway and may lead to underestimation of the true genetic burden. Fourth, the male subgroup (n = 76) had limited power (~55% for OR = 1.88, α = 0.05); thus, the non-significant result in males may reflect insufficient power. Approximately 150–180 male cases would be required for 80% power (**Supplementary Figure 2**).

Finally, these findings are associative and do not establish causality. Future studies integrating phosphoproteomics, transcriptomics, and computational approaches (e.g., variant effect prediction, pathway modeling, and single-cell polygenic risk analyses) are needed to clarify the functional impact of these variants in microscopic polyangiitis. In this regard, emerging computational frameworks such as single-cell Latent-variable Model (scLM) (45) and xSiGra (46)offer promising strategies for dissecting gene–cell interactions in complex immune microenvironments.

## Data Availability

The data presented in this study are not publicly available due to ethical and institutional restrictions related to patient privacy and data protection regulations. De-identified data supporting the findings of this study may be made available from the corresponding author upon reasonable request, subject to approval by the institutional ethics committee and in accordance with institutional data governance policies.

## Supplementary material

Supplementary Figure S1 Principal component analysis (PCA) of study participants based on four PI3K–AKT–mTOR pathway SNPs. PCA was conducted using standardized additive genotype data for four SNPs (PIK3CA rs1607237, AKT1 rs2498786, MTOR rs2295080, and MTOR rs1057079). Each point represents one individual. Panel **(A)** includes all groups: MPA patients (red, n = 202), 1000 Genomes controls (blue, n = 387), and hospital-based controls (green, n = 208); Panel **(B)** shows controls only. Ellipses denote 95% confidence regions (2 SD). The variance explained by each principal component is indicated on the axes. No clear clustering by sample source was observed, suggesting minimal population stratification. Ten samples with missing genotypes were excluded.

Supplementary Figure S2 Post hoc power analysis for sex-stratified comparisons. Statistical power (1−β) was estimated as a function of the true odds ratio (OR) for high vs. low genetic burden (two-sided α = 0.05). In females (126 cases, 330 controls; control high-burden frequency 14.6%), power reached 86% at the observed OR of 2.09 (red solid line). In males (76 cases, 265 controls; 16.6%), power was 55% at an OR of 1.88 (blue dashed line). The horizontal dashed line denotes 80% power. Results for males should be interpreted cautiously due to limited power.
