# Supplementary material for: Genetic burden across core genes of the PI3K–AKT–mTOR pathway is associated with susceptibility to microscopic polyangiitis: a Chinese cohort study
Source: Front Immunol. 2026 Apr 23;17:1807517. doi: 10.3389/fimmu.2026.1807517 (PMC13149408; doi:10.3389/fimmu.2026.1807517)
Supplement: Supplementary Figure S1 — Principal component analysis (PCA) of study participants based on four PI3K–AKT–mTOR pathway SNPs. PCA was conducted using standardized additive genotype data for four SNPs (PIK3CA rs1607237, AKT1 rs2498786, MTOR rs2295080, and MTOR rs1057079). Each point represents one individual. Panel (A) includes all groups: MPA patients (red, n = 202), 1000 Genomes controls (blue, n = 387), and hospital-based controls (green, n = 208); Panel (B) shows controls only. Ellipses denote 95% confidence regions (2 SD). The variance explained by each principal component is indicated on the axes. No clear clustering by sample source was observed, suggesting minimal population stratification. Ten samples with missing genotypes were excluded. [file Table1.docx]

**Supplementary Table S1 Genotype Homogeneity Tests (Hospital vs. 1000 Genomes controls):**

| SNP | Chi-square | df | P (raw) | P (FDR-BH) |
| --- | --- | --- | --- | --- |
| PIK3CA rs1607237 | 1.977 | 2 | 0.372 | 0.691 |
| AKT1  rs2498786 | 8.850 | 2 | 0.012 | 0.048* |
| MTOR rs2295080 | 1.315 | 2 | 0.518 | 0.691 |
| MTOR rs1057079 | 0.026 | 2 | 0.987 | 0.987 |

**Supplementary Table S2 *Single-variant associations of PI3K–AKT–mTOR pathway SNPs with MPA risk***

| SNP | Genetic model | Genotype | MPA  n (%) | Control  n (%) | OR (95% CI) | P (FDR-BH) |
| --- | --- | --- | --- | --- | --- | --- |
| PIK3CA rs1607237 | Codominant | C/C | 273 (46.0) | 116 (60.1) | 1.00 | **0.0120*** |
|  |  | C/T | 244 (41.1) | 57 (29.5) | 0.55 (0.39–0.79) |  |
|  |  | T/T | 77 (13.0) | 20 (10.4) | 0.61 (0.36–1.05) |  |
|  | Dominant | C/C | 273 (46.0) | 116 (60.1) | 1.00 | **0.0080**** |
|  |  | C/T–T/T | 321 (54.0) | 77 (39.9) | 0.57 (0.41–0.79) |  |
|  | Log-additive | - | - | - | 0.69 (0.54–0.89) | **0.0120*** |
| AKT1 rs2498786 | Codominant | C/C | 356 (59.8) | 145 (71.8) | 1.00 | **0.0080**** |
|  |  | C/G | 205 (34.5) | 54 (26.7) | 0.64 (0.45–0.92) |  |
|  |  | G/G | 34 (5.7) | 3 (1.5) | 0.22 (0.07–0.71) |  |
|  | Dominant | C/C | 356 (59.8) | 145 (71.8) | 1.00 | **0.0095**** |
|  |  | C/G–G/G | 239 (40.2) | 57 (28.2) | 0.58 (0.41–0.82) |  |
|  | Log-additive | - | - | - | 0.59 (0.43–0.80) | **0.0080**** |
| MTOR rs2295080 | Codominant | T/T | 343 (57.6) | 115 (56.9) | 1.00 | 0.1400 |
|  |  | T/G | 218 (36.6) | 82 (40.6) | 1.14 (0.82–1.59) |  |
|  |  | G/G | 34 (5.7) | 5 (2.5) | 0.43 (0.16–1.12) |  |
| MTOR rs1057079 | Codominant | T/T | 365 (61.3) | 120 (59.4) | 1.00 | **0.0172*** |
|  |  | T/C | 197 (33.1) | 79 (39.1) | 1.25 (0.89–1.74) |  |
|  |  | C/C | 33 (5.5) | 3 (1.5) | 0.26 (0.08–0.87) |  |

Footnote: Odds ratios (ORs) and 95% confidence intervals (CIs) were estimated using logistic regression adjusted for sex. *****, P (FDR-BH) ＜0.05. ******, P (FDR-BH) ＜0.01.

**Supplementary Table S3 Sex-interaction analysis of PI3K–AKT–mTOR polymorphisms and risk of MPA in Codominant model**

| SNP | Sex | OR (95% CI) | P- Interaction | P- Interaction (FDR-BH) |
| --- | --- | --- | --- | --- |
| PIK3CA rs1607237 | Female | 0.60 (0.38–0.96) | 0.031 | 0.062 |
|  | Male | 0.48 (0.27–0.86) | 0.013 | 0.065 |
| AKT1  rs2498786 | Female | 0.39 (0.24–0.64) | <0.0001 | **0.0007***** |
|  | Male | 1.27 (0.74–2.17) | 0.380 | 0.480 |
| MTOR rs2295080 | Female | 1.78 (1.16–2.72) | 0.008 | **0.032*** |
|  | Male | 0.57 (0.33–0.98) | 0.043 | 0.086 |
| MTOR rs1057079 | Female | 1.81 (1.18–2.78) | 0.007 | **0.028*** |
|  | Male | 0.72 (0.42–1.24) | 0.240 | 0.320 |

Footnote: Odds ratios (ORs) and 95% confidence intervals (CIs) were estimated using logistic regression adjusted for sex. *****, P (FDR-BH) ＜0.05. *******, P (FDR-BH) ＜0.001.

**Supplementary Table S4 SNP Association Analysis in Females (Crude Analysis, n=456)**

| SNP | Genetic model | Genotype | OR (95% CI) | P (raw) | P (FDR-BH) |
| --- | --- | --- | --- | --- | --- |
| PIK3CA rs1607237 | Codominant | C/C vs. C/T vs. T/T | 0.60 (0.38–0.96) / 0.60 (0.30–1.20) | 0.063 | 0.089 |
|  | Dominant | C/C vs. C/T–T/T | 0.60 (0.39–0.92) | **0.019** | **0.032*** |
|  | Overdominant | C/C–T/T vs. C/T | 0.66 (0.42–1.04) | 0.070 | 0.089 |
|  | Recessive | C/C–C/T vs. T/T | 0.73 (0.37–1.43) | 0.340 | 0.340 |
|  | Log-additive | C→T | 0.71 (0.52–0.98) | **0.031** | **0.044*** |
| AKT1 rs2498786 | Codominant | C/C vs. C/G vs. G/G | 0.39 (0.24–0.64) / 0.20 (0.05–0.88) | **0.0001** | **0.0005***** |
|  | Dominant | C/C vs. C/G–G/G | 0.37 (0.23–0.59) | **<0.0001** | **<0.0001***** |
|  | Overdominant | C/C–G/G vs. C/G | 0.43 (0.26–0.69) | **0.0003** | **0.0007***** |
|  | Recessive | C/C–C/G vs. G/G | 0.26 (0.06–1.15) | **0.036** | **0.046*** |
|  | Log-additive | C→G | 0.41 (0.27–0.63) | **<0.0001** | **<0.0001***** |
| MTOR rs2295080 | Codominant | T/T vs. T/G vs. G/G | 1.78 (1.16–2.72) / 0.12 (0.02–0.92) | **0.0001** | **0.0022**** |
|  | Dominant | T/T vs. T/G–G/G | 1.45 (0.96–2.20) | 0.077 | 0.089 |
|  | Overdominant | T/T–G/G vs. T/G | 1.97 (1.29–3.00) | **0.0017** | **0.0022**** |
|  | Recessive | T/T–T/G vs. G/G | 0.10 (0.01–0.73) | **0.0011** | **0.0018**** |
|  | Log-additive | T→G | 1.07 (0.76–1.50) | 0.710 | 0.710 |
| MTOR rs1057079 | Codominant | T/T vs. T/C vs. C/C | 1.81 (1.18–2.78) / 0.12 (0.02–0.87) | **0.0001** | **0.0022**** |
|  | Dominant | T/T vs. T/C–C/C | 1.43 (0.94–2.17) | 0.096 | 0.103 |
|  | Overdominant | T/T–C/C vs. T/C | 2.00 (1.30–3.07) | **0.0016** | **0.0022**** |
|  | Recessive | T/T–T/C vs. C/C | 0.09 (0.01–0.70) | **0.0008** | **0.0016**** |
|  | Log-additive | T→C | 1.04 (0.74–1.46) | 0.820 | 0.820 |

Footnote: Odds ratios (ORs) and 95% confidence intervals (CIs) were estimated using logistic regression adjusted for sex. *****, P (FDR-BH) ＜0.05. ******, P (FDR-BH) ＜0.01. *******, P (FDR-BH) ＜0.001.

**Supplementary Table S5 SNP Association Analysis in Males (Crude Analysis, n=341)**

| SNP | Genetic model | Genotype | OR (95% CI) | P (raw) | P (FDR-BH) |
| --- | --- | --- | --- | --- | --- |
| PIK3CA rs1607237 | Codominant | C/C vs. C/T vs. T/T | 0.48 (0.27–0.86) / 0.64 (0.27–1.48) | 0.038 | 0.095 |
|  | Dominant | C/C vs. C/T–T/T | 0.52 (0.31–0.88) | 0.013 | 0.065 |
|  | Overdominant | C/C–T/T vs. C/T | 0.52 (0.30–0.92) | 0.021 | 0.070 |
|  | Recessive | C/C–C/T vs. T/T | 0.85 (0.38–1.93) | 0.700 | 0.700 |
|  | Log-additive | Per allele (C→T) | 0.67 (0.45–1.00) | 0.043 | 0.086 |
| AKT1 rs2498786 | Codominant | C/C vs. C/G vs. G/G | 1.27 (0.74–2.17) / 0.24 (0.03–1.87) | 0.140 | 0.200 |
|  | Dominant | C/C vs. C/G–G/G | 1.10 (0.65–1.87) | 0.710 | 0.791 |
|  | Overdominant | C/C–G/G vs. C/G | 1.35 (0.79–2.30) | 0.280 | 0.280 |
|  | Recessive | C/C–C/G vs. G/G | 0.22 (0.03–1.71) | 0.074 | 0.148 |
|  | Log-additive | Per allele (C→G) | 0.94 (0.60–1.47) | 0.790 | 0.791 |
| MTOR rs2295080 | Codominant | T/T vs. T/G vs. G/G | 0.57 (0.33–0.98) / 1.27 (0.37–4.31) | 0.094 | 0.157 |
|  | Dominant | T/T vs. T/G–G/G | 0.62 (0.36–1.05) | 0.070 | 0.140 |
|  | Overdominant | T/T–G/G vs. T/G | 0.56 (0.32–0.96) | 0.032 | 0.080 |
|  | Recessive | T/T–T/G vs. G/G | 1.58 (0.47–5.28) | 0.470 | 0.588 |
|  | Log-additive | Per allele (T→G) | 0.73 (0.46–1.17) | 0.180 | 0.225 |
| MTOR rs1057079 | Codominant | T/T vs. T/C vs. C/C | 0.72 (0.42–1.24) / 0.88 (0.18–4.38) | 0.490 | 0.490 |
|  | Dominant | T/T vs. T/C–C/C | 0.73 (0.43–1.24) | 0.240 | 0.267 |
|  | Overdominant | T/T–C/C vs. T/C | 0.72 (0.42–1.24) | 0.230 | 0.267 |
|  | Recessive | T/T–T/C vs. C/C | 1.00 (0.20–4.90) | 1.000 | 1.000 |
|  | Log-additive | Per allele (T→C) | 0.77 (0.48–1.25) | 0.290 | 0.322 |

Footnote: Odds ratios (ORs) and 95% confidence intervals (CIs) were estimated using logistic regression adjusted for sex.

**Supplementary Table S6 SNP Association with MPO-ANCA Positivity (Adjusted for Sex, n=727-734)**

| SNP | Genetic model | Genotype | OR (95% CI) | P (raw) | P (FDR-BH) |
| --- | --- | --- | --- | --- | --- |
| PIK3CA rs1607237 | Codominant | C/C vs. C/T vs. T/T | 0.64 (0.42–0.96) / 0.65 (0.35–1.22) | 0.070 | 0.117 |
|  | Dominant | C/C vs. C/T–T/T | 0.64 (0.44–0.94) | **0.021** | **0.048*** |
|  | Overdominant | C/C–T/T vs. C/T | 0.69 (0.46–1.03) | 0.064 | 0.117 |
|  | Recessive | C/C–C/T vs. T/T | 0.79 (0.43–1.44) | 0.430 | 0.537 |
|  | Log-additive | Per allele (C→T) | 0.74 (0.56–0.99) | 0.039 | 0.078 |
| AKT1 rs2498786 | Codominant | C/C vs. C/G vs. G/G | 0.61 (0.40–0.92) / 0.20 (0.05–0.86) | **0.0032** | **0.013*** |
|  | Dominant | C/C vs. C/G–G/G | 0.55 (0.37–0.83) | **0.0032** | **0.013*** |
|  | Overdominant | C/C–G/G vs. C/G | 0.65 (0.43–0.99) | 0.041 | 0.078 |
|  | Recessive | C/C–C/G vs. G/G | 0.24 (0.06–1.01) | 0.016 | 0.043***** |
|  | Log-additive | Per allele (C→G) | 0.56 (0.39–0.80) | **0.001** | **0.010*** |
| MTOR rs2295080 | Codominant | T/T vs. T/G vs. G/G | 1.05 (0.71–1.54) / 0.47 (0.16–1.38) | 0.290 | 0.414 |
|  | Dominant | T/T vs. T/G–G/G | 0.97 (0.66–1.41) | 0.860 | 0.860 |
|  | Overdominant | T/T–G/G vs. T/G | 1.10 (0.75–1.61) | 0.620 | 0.689 |
|  | Recessive | T/T–T/G vs. G/G | 0.47 (0.16–1.34) | 0.120 | 0.171 |
|  | Log-additive | Per allele (T→G) | 0.89 (0.65–1.23) | 0.480 | 0.600 |
| MTOR rs1057079 | Codominant | T/T vs. T/C vs. C/C | 1.25 (0.85–1.84) / 0.25 (0.06–1.05) | **0.024** | **0.048*** |
|  | Dominant | T/T vs. T/C–C/C | 1.09 (0.75–1.59) | 0.650 | 0.722 |
|  | Overdominant | T/T–C/C vs. T/C | 1.34 (0.91–1.96) | 0.140 | 0.200 |
|  | Recessive | T/T–T/C vs. C/C | 0.23 (0.05–0.96) | **0.013** | **0.043*** |
|  | Log-additive | Per allele (T→C) | 0.93 (0.68–1.28) | 0.660 | 0.733 |

Footnote: Odds ratios (ORs) and 95% confidence intervals (CIs) were estimated using logistic regression adjusted for sex. *****, P (FDR-BH) ＜0.05.

**Supplementary Table S7. Sensitivity Analysis of Genetic Burden and MPA Risk Restricted to Hospital-Based Controls (Adjusted for Age and Sex)**

| Genetic Burden | MPA (n = 202) | | Hospital Controls  (n = 209) | OR  (95% CI)† | P value |
| --- | --- | --- | --- | --- | --- |
| Low (reference) | 52 (25.7%) | | 79 (37.8%) | 1.00 | — |
| Intermediate | 92 (45.5%) | | 96 (45.9%) | 1.46 (0.92–2.31) | 0.108 |
| High | 49 (24.3%) | | 34 (16.3%) | 2.19 (1.26–3.81) | **0.005**** |
| P for trend | | **0.006**** | | | |

Footnote: Sensitivity analysis using only hospital-based controls (n = 209), excluding 1000 Genomes controls. Risk allele definitions and burden categories were identical to the primary analysis. Results were consistent, with the high-burden group remaining significantly associated with MPA risk. Odds ratios (ORs) and 95% confidence intervals (CIs) were estimated using logistic regression adjusted for age and sex. **, P ＜0.01.
