# Supplementary figures and images for: Genetic burden across core genes of the PI3K–AKT–mTOR pathway is associated with susceptibility to microscopic polyangiitis: a Chinese cohort study
Source: Front Immunol. 2026 Apr 23;17:1807517. doi: 10.3389/fimmu.2026.1807517 (PMC13149408; doi:10.3389/fimmu.2026.1807517)

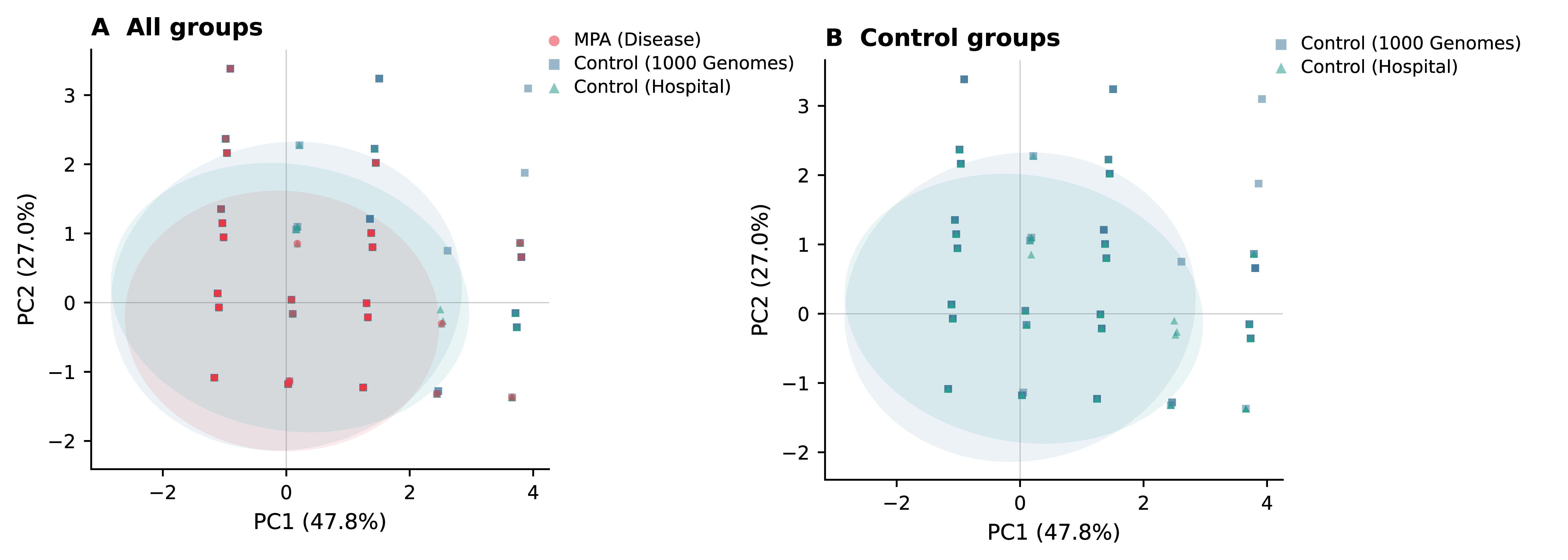

Supplement: Supplementary file 2 [file Image1.tiff]
